# Hall technology and conventional PMC technology: Progress and remaining challenge in extensive caries treatment

**DOI:** 10.1097/MD.0000000000043130

**Published:** 2025-07-04

**Authors:** Xue Zeng, Pengcheng He, Bingyao Kang

**Affiliations:** a Department of Pediatric Outpatient Nursing, West China Second University Hospital, Sichuan University, Chengdu, Sichuan, China; b Key Laboratory of Birth Defects and Related Diseases of Women and Children (Sichuan University), Ministry of Education, Chengdu, Sichuan, China; c Department of Pediatric Dentistry, West China Second University Hospital, Sichuan University, Chengdu, Sichuan, China.

**Keywords:** deciduous molars, Hall technology, oral health of children, preformed mental crown technology

## Abstract

Preformed metal crown restoration technology is widely used in the treatment of large area caries of deciduous molars, which can not only restore the tooth shape but also restore the chewing function of children. Preformed mental crown restoration technology includes conventional preformed mental crown technology and Hall technology. In this paper, the application scope, clinical curative effect, and research progress of clinical-related problems of premolar metal crown in large area caries of children were summarized.

## 1. Introduction

Dental caries is the most common chronic infectious disease in children. If not treated in time, it can lead to pulpitis, periapical inflammation, and even affect the normal development and eruption of the permanent tooth,^[1]^ especially caries in the molar teeth, which can lead to the loss of tooth space and the reduction of chewing function, which seriously affects the physical and mental development of the children.^[2]^ Currently, the treatment of caries in molar teeth includes filling treatment and preformed mental crowns (PMC), which have been widely used in the treatment of extensive caries in molar teeth because of their ability to restore the shape of the child’s teeth and the function of mastication and has been recognized as the best treatment for large caries in molar teeth by the International Association of Pediatric Dentistry. The International Association of Pediatric Dentistry recognizes PMC as one of the preferred treatments for caries in molar teeth.^[3]^

PMC is a metal restoration prefabricated from stainless steel that simulates the morphology of a molar tooth. At the beginning of the 21st century, PMC was used in the UK as a restorative treatment for large caries or endodontically treated teeth^[4]^ and has been gradually popularized in pediatric dentistry abroad. Since conventional PMC technology requires tooth preparation, some children who suffer from dental fear are not able to cooperate with the completion of the relevant treatment.^[5]^ Therefore, in the 1980s, Norna Hall proposed to select the appropriate size of preformed crowns and cement them directly to the decayed molar teeth after dental cleaning without tooth preparation and decalcification and named it Hall technology. It has been found that the occlusion of a child is elevated after treatment because no tooth preparation or decay removal was performed, but with occlusal adjustment of the child, it can be restored to the preoperative occlusal state.^[6]^ So is there any difference between the conventional PMC technology and the Hall technology in terms of indications, clinical efficacy, and clinically relevant issues? In this paper, we report on the research progress of the above issues, to provide a reference for the selection of protocols for the treatment of caries in children.

## 2. Principle and indication of treatment

**Figure 1.**
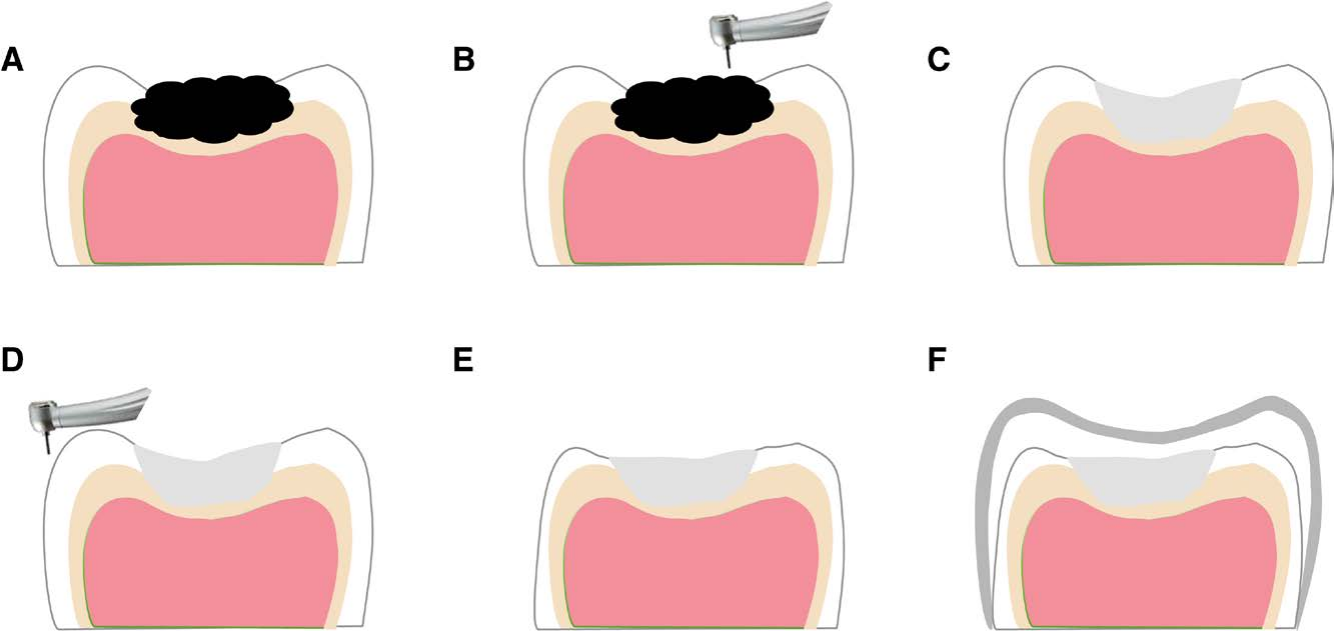
Conventional PMC technology. PMC = preformed metal crown.

**Figure 2.**
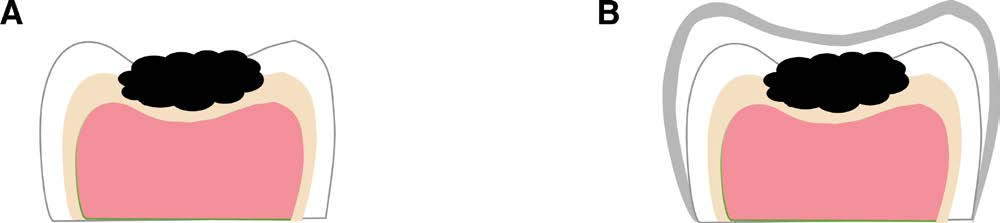
Hall technology.

The conventional PMC technology is mainly for large defective teeth, after tooth preparation, through the bonding of PMC to make it a whole, enhance its retention and resistance to prolong its service life to the replacement of permanent teeth^[7]^ (Fig. 1A–G); compared to the conventional PMC technology, Hall technology did not carry out the tooth preparation and decalcification (Fig. 2A and B). After the disinfection and bonding the suitable PMC, saliva, and cariogenic micro-environment are isolated, to achieve the role of slowing down or even stopping the development of caries, while restoring its morphology and function.^[8]^ Both of the 2 treatments get the familiar cementation process: after choosing a suitable PMC, gauze is used for airway protection and protecting the tooth from saliva. Then fill the crown with cement (usually glass ionomer cement is recommended) and seat it over the tooth. In this case, let the child bite to seat the crown with cotton wool to help distribute the force. The gingiva is blanching as the crown is sitting slightly subgingivally, further improving the seal and preventing the lesion from progressing.

The indications for conventional PMC technology mainly include: ① restoration of deciduous or young permanent teeth with large loss of tooth tissue due to caries, dystrophy or abrasion; ② restoration of deciduous and young permanent teeth at risk of crown fracture after endodontic treatment; ③ retention of bad habit orthodontic appliances; ④ retention of a variety of fixed gap maintainers.^[9]^ By reviewing the literature, the indications for Hall technology mainly include: ① high loss of tooth tissue due to caries, dystrophy, or abrasion; ② caries not involving the pulp cavity on imaging. It can be seen that the indications for the Hall technology are mainly for the treatment of molar teeth without pulpal involvement, and for those teeth with symptoms of pulpitis or other therapeutic purposes, conventional PMC restorations are considered, and the main purpose of using the Hall technique is to simplify the operation process and improve the degree of cooperation of the child.^[10]^

## 3. Prognosis

Traditional PMC technology will be performed after complete decalcification and filling, which can remove all existing caries and prevent further development of caries, but this will also increase the discomfort of the children during the treatment, and increase the risk of accidental pulp penetration leading to treatment failure. The Hall technology, on the other hand, omits the step of decalcification and slows or even stops the further development of caries by physically isolating the saliva from the cariogenic microenvironment of the oral cavity. Because of the lack of decalcification, many clinicians are skeptical about whether caries will develop further and whether the pulp will be involved, and because of the unfamiliarity with the operation, this makes some clinicians not use it as the first choice for the treatment of extensive caries in children’s molar teeth.^[11]^

Ludwig et al^[12]^ 2014 followed up children in the Hall technology group as well as in the conventional PMC technology group by imaging and clinical examination for up to 5 years, with each patient followed up for at least 6 months or until discontinuation at failure. The results showed that the average follow-up time was 15 months in the Hall technology group, with a success rate of 97%, and 53 months in the conventional PMC technology group, with a success rate of 94%. Two of the failures in the Hall technique group were due to periapical abscesses, and 7 failures in the conventional PMC technology group were due to infections in 5 cases and loosening and detachment of the preformed crowns in 2 cases. Elamin et al^[13]^ in a two-year follow-up survey, found that the success rate of both treatment technologies could reach >90% (Hall technique: 94.5% conventional PMC: 96%) with no statistically significant difference. There was no statistically significant difference between the 2 techniques (Hall group: 94.5%, conventional PMC group: 96%). It is easy to see that both the Hall technology and the conventional PMC technology have a favorable prognosis in terms of success rate. The key factor for the success of both is the presence of perfect marginal sealing to prevent secondary caries or caries progression, thus preserving the affected teeth until the replacement. It has also been found that the average length of time for conventional PMC restorations is 3 times longer than that of Hall technology, and due to the longer treatment operation time, children’s cooperation decreases, although Hall technology may sometimes need to place the pressure ring in advance due to insufficient space, this is not a continuous operation in the mouth and does not cause significant discomfort to the child.^[14]^ At the same time, some scholars^[15]^ compared the Hall technology with the traditional filling technology in terms of success rate, cost, and prognosis, and found that the Hall technique was better than the traditional filling technology in terms of success rate and prognosis, and the cost of its follow-up due to fewer complications at the later stage of the treatment of secondary caries, endodontitis, etc, the expenditure is less. Therefore, it is easy to see that the Hall technology is better in terms of success rate, prognosis, and profitability.

## 4. Effect on occlusion

Childhood is undergoing rapid development, and the jaws and temporomandibular joints (TMJs) are in the process of remodeling, which facilitates the replacement of permanent teeth and the construction of jaws, which also provides conditions for occlusal management and early orthodontic treatment in children.^[16]^ Therefore, children have a better ability to compensate for occlusal elevation during this period. PMC is a prefabricated restoration, after restoration, occlusal elevation occurs in the child’s occlusion. Although the traditional PMC technology has lowered the jaw in advance, in some special cases, such as the treatment of caries in 8 molar teeth under general anesthesia, the occlusion of the child cannot be fully assessed under general anesthesia and the PMC itself can cause an occlusal high spot, which will result in a significant elevation of the occlusion of the child. The Hall technology also results in a more pronounced change in occlusal due to the lack of tooth preparation, with some studies suggesting an average occlusal elevation of 2 to 2.5 mm after treatment with the Hall technology.^[17]^

Occlusal elevation in children has been studied through changes in the cusp relationship and vertical dimension of occlusion (VDO). Shih YC et al^[18]^ found that VDO values returned to the preoperative state by approximately 1 month postoperatively by measuring the cusp occlusal relationship in 39 children who were treated with conventional PMC technology on 8 molar teeth under general anesthesia. Van et al^[17]^ recorded the cusp relationships of 48 children treated with the Hall technology by measuring the cusp relationship and found that the cusp relationship of the children decreased from 2.45 mm to 0.54 mm immediately after treatment with the Hall technology, and then their cusp relationship returned to the preoperative status after 8 weeks. Nair et al indirectly reflected the change in overdenture by measuring the VDO value and found that there was also an increase in the immediate postoperative period, but the occlusion gradually decreased to the preoperative level after several weeks as the postoperative period increased.^[19]^ It is easy to see that the occurrence of occlusal elevation after treatment with PMC will be restored to the preoperative state by occlusal adjustments.

The specific mechanisms of occlusal remodeling in children are still unknown. Some researchers have proposed that the appearance of occlusal high points leads to an increase in masticatory pressure on a single tooth, which increases the force on the periodontal tissues in the apical region and activates the osteoclasts in the apical region, leading to resorption of alveolar bone in the apical region and movement of the tooth root toward the root to complete occlusal remodeling.^[20]^ Some researchers have also measured the crown heights of the affected teeth treated with the Hall technology as well as the opposing dentition, and found that at 30 days postoperative, the crown heights of the affected teeth treated with the Hall technology and their opposing dentition decreased, which was considered to be a result of the co-adjustment of the affected teeth and the opposing dentition during occlusal remodeling.^[21]^ Meanwhile, during the postoperative follow-up period, the jaws were also in the process of growth and development, which should not be ignored for the occlusal height adjustment.^[22]^

## 5. Effect on TMJ

The etiology of temporomandibular disorder (TMD) is unclear, but it has been associated with a variety of pathogenic factors, including intra- and extra-articular trauma, psychosomatic factors, and malocclusion.^[23]^ TMD is common in females aged 20 to 40 years old, and is characterized by symptoms such as pain in the joint area, rattling, and jaw dyskinesia.The prevalence of TMD is relatively low in younger children, and the prevalence is 7.3% to 30.4% in children aged 10 to 19 years old.^[24]^ It is hypothesized that the reason may be that the joints are in the process of developmental remodeling at the young age. The prevalence of TMD in younger children is low, ranging from 7.3% to 30.4% in children aged 10 to 19 years, which is presumed to be because the joints of younger children are in the process of developmental remodeling, and the joints are gradually developing and molding as they grow older.^[24]^ Since PMC inevitably leads to occlusal changes in children, whether treated by conventional PMC technology or Hall technology, which results in changes in condylar position and joint muscles, they become a potential risk factor for TMD in children.

Due to ethical reasons, clinical trials for the routine examination of TMJ can not be X-ray filming, mostly based on questionnaires and clinical examination records, the current commonly used TMJ assessment methods for the modified Helkimo’s Clinical Impairment Index (Di)^[25]^ and questionnaires were recorded and evaluated respectively on the clinical symptoms and the patient’s subjective feelings, Di mainly includes the patient’s opening degree, opening deviation, and TMJ dysfunction were evaluated and graded. Shih YC^[18]^ et al performed TMJ examinations on 39 children who were treated with the traditional PMC technology for 8 molar teeth at one time under general anesthesia at preoperatively, 1 week postoperatively, 1 month postoperatively, 3 months postoperatively, and 6 months postoperatively, and the results showed that only 3 children showed mild dysfunction postoperatively, which disappeared as the postoperative time prolongation, and 4 children had subjective discomfort at 1 week postoperatively which disappeared after 1 month postoperatively. Kaya et al^[26]^ on the other hand, examined and evaluated the joints of 39 children who were treated with Hal technology for a single molar tooth at 0, 1, 3, 6, and 12 months postoperatively, and found that there was no statistically significant difference in the joint data compared to the preoperative period. Innes,^[27]^ Joseph^[22]^ and others also followed up with children treated with Hall technology by questionnaire and none of them were found to have irreversible positive signs.

The TMJ consists of the articular fossa, articular disc, and condylar process.^[28]^ The morphology of the TMJ is not fully stereotyped at birth, but rather, during the 2 rapid growth periods of 5 to 10 and 10 to 15 years of age,^[29]^ the TMJ serves as a germinal center to promote the maxillofacial development, while gradually remodeling itself to finalize its shape.^[30]^ At birth, the articular fossa is flat and gradually deepens with age under the action of pulling the surrounding muscles and the pressure of mastication to form an articular fossa with a certain depth.^[31]^ Most of the condylar cartilage is replaced by intraperitoneal osteogenesis at birth, but a small portion of the condylar cartilage undergoes slow remodeling until adulthood, which is important for maintaining a stable position of the condyle about the temporal bone. The shape of the articular tuberosity begins to develop at the age of 3 years, and the final adult shape is achieved through masticatory forces and biological adjustments until the age of about 12 years. It is due to the high plasticity of the developmental adjustments of the TMJ until the age of 12 years that it is highly adaptable to occlusal changes.^[32]^ Also the TMJ itself is highly adaptable. A padded occlusion is commonly used in medicine for the treatment of severe bruxism and orthodontic patients,^[21]^ and it has been shown that the VDO value can be increased up to 5 mm within the range of physiologic adaptation.^[33]^ Therefore, in the children treated with PMC, although the occlusal contact points and VDO values of the children were changed by both the traditional PMC technology and the Hall technology, the occlusal contact points and VDO values of the children did not exceed the physiological adaptive range of the TMJs, and at the same time, they were at the peak of the TMJs’ growth and development, which was more favorable for the children to adapt to their own adaptations and adjustments.

## 6. Impact of periodontal health

Plaque and tartar buildup, poor oral hygiene habits, and incorrect brushing may lead to periodontal disease.^[34]^ PMC without cervical preparation and Hall technology leads to early contact and increased occlusal forces on a single tooth are potential causative factors for periodontal inflammation in children after treatment. Shashikala Prabhu^[35]^ et al followed up on the gingival health and oral hygiene of 60 children undergoing PMC restorations at month 3 and month 6 after treatment. The results showed that the gingival and oral hygiene status of the restored teeth with metal preformed crowns was not significantly abnormal compared to the restored teeth on the opposite side of the mouth without restorative treatment. Several studies have reported that the degree of gingival extension and proper fit of metal prefabricated crowns do not lead to periodontal problems,^[36]^ but when the metal prefabricated crowns are not properly sized for the tooth, causing compression of the gingival tissues, and are in too tight a contact with the neighboring teeth, they can lead to gingival inflammation and alveolar bone resorption.^[37]^ The treatment of the margin of the crown is also very important for periodontal health, as a poorly fitting margin can lead to the buildup of subgingival plaque and a crown margin that is not polished can act as a mechanical irritant leading to periodontal problems.^[38]^ Mild inflammation of the gingival tissues may occur within a short period of time after metal-preformed crown treatment, which may be related to the small amount of adhesive remaining in the gingival sulcus and irritation of the PMC margins, and the inflammation may subside on its own when the body adjusts to it.

Periodontal potential, also known as periodontal reserve force, refers to the fact that during normal masticatory movements, the occlusal force for chewing food is approximately half of the force that the periodontal tissues are able to support, and the other half of the supportive capacity is reserved in the periodontal tissues.^[39]^ Therefore if the increase in the occlusal force of the dental tissues is within the appropriate range, it will not cause damage to the periodontal tissues.Gallagher et al^[22]^ measured the occlusal force of teeth treated with PMC restorations and found that although the percentage of the occlusal force was elevated on the treated side, the percentage of the occlusal force returned to the normal level with the increase in the postoperative follow-up. Nair et al,^[19]^ on the other hand, measured the occlusal force of the teeth of children after Hall technology treatment and found that the occlusal force of single teeth increased after Hall technology treatment, but the occlusal force of the whole mouth decreased, and with occlusal adjustment, the occlusal force of the treated tooth position and the occlusal force of the whole mouth returned to the preoperative level. PMC restorative treatment after PMC restoration treatment leads to a temporary increase in the occlusal force, but it still remains within the normal range, and the periodontal tissues of the child have a strong adaptive remodeling The periodontal tissues of the child are capable of adapting to remodeling, so the use of a suitable metal prefabricated crown with a perfect cervical margin will not lead to periodontal problems.

## 7. Zirconia crowns for the primary tooth

Along with advance of manufacturing technology, apart from PMC, prefabricated zirconia crowns are gradually used in primary teeth. Compared with PMC, the main advantages of zirconia crowns are their esthetically excellent appearance alongside their durability, but the zirconia crowns require more aggressive tooth reduction and are more expensive than conventional PMC technology.^[40,41]^ Geduk et al^[42]^ found that zirconia crowns showed high clinical retention rates in children compared with PMCs with conventional technology. Walia et al^[43]^ compared the clinical outcomes of 3 aesthetic full-coronal restorations with conventional technology (composite strip crowns, PMCs, and zirconia crowns), and the results showed that zirconia crowns are highly retentive and biocompatible but cause low grade of abrasion of their opposing natural dentition at the 6-month follow-up than another 2. Some studies^[44,45]^ also reported that zirconia crowns had lower plaque accumulation and better gingival health than PMCs due to their highly polished surface. However, zirconia crowns have not been reported to use in Hall technology, this may be related to its low ductility.

## 8. Conclusion

PMC restorative technology include Hall technology and conventional PMC technology, both of which can achieve good clinical results, and due to its good edge closure, secondary caries and filling loss are rare. Although early contact and occlusal changes may occur temporarily after treatment, with occlusal reconstruction and body adaptation, the occlusion will gradually return to the normal level without affecting the TMJ and periodontal tissues.

## Author contributions

**Conceptualization:** Bingyao Kang.

**Investigation:** Pengcheng He.

**Visualization:** Pengcheng He.

**Writing – review & editing:** Xue Zeng.
